# Venous thromboembolism prophylaxis in adults hospitalised for psychiatric illness: an evidence-based clinical practice guideline developed using GRADE

**DOI:** 10.1007/s11096-025-02072-1

**Published:** 2026-01-16

**Authors:** Audrey Purcell, Fionnuala Ní Áinle, Beverley J. Hunt, Aurelien Delluc, Lara Roberts, Josie Jenkinson, Jennifer Hoblyn, Dolores Keating, Aoife Carolan, Eric Roche, Kathy Morgan, Richard Duffy, Sarah Garvey, Joanne Flood, Ann Marie O′ Neill, Arnav Agarwal

**Affiliations:** 1 St John of God University Hospital, Dublin, Ireland; 2 School of Pharmacy and Biomolecular Science, Royal College of Surgeons, Dublin, Ireland; 3 National Clinical Programme in Venous Thromboembolism, Health Services Executive, Dr Steeven’s Hospital, Dublin, Ireland; 4 School of Medicine, University College Dublin, Dublin, Ireland; 5 Thrombosis and Haemostasis, Guy′s and St Thomas′ NHS Foundation Trust, London, UK; 6 Faculty of Medicine, Department of Medicine, Division of Hematology, University of Ottawa, Ottawa, Canada; 7 Department of Haematological Medicine, King’s College Hospital NHS Foundation Trust, London, UK; 8 Surrey and Borders Partnership, NHS Foundation Trust, Leatherhead, UK; 9 Department of Brain Sciences, Imperial College London, London, UK; 10 Faculty of Old Age Psychiatry, Royal College of Psychiatrists, London, UK; 11 Cluain Mhuire Community Mental Health Service, Dublin, Ireland; 12 School of Medicine, RCSI University of Medicine and Health Sciences, Dublin, Ireland; 13 Pennine Care NHS Foundation Trust, Ashton-under-Lyne, UK; 14 Rotunda Hospital, Dublin, Ireland; 15 Beaumont Hospital, Dublin, Ireland; 16 Mental Health Services for Older People, North Dublin, Ireland; 17 Patient Partner, Dublin, Ireland; 18 Division of General Internal Medicine, Department of Medicine, University of Alberta, 5-112 Clinical Sciences Building, 11304 – 83 Ave NW, Edmonton, AB T6G 2B7 Canada; 19 Department of Health Research Methods, Evidence and Impact, McMaster University, Hamilton, ON Canada; 20 Division of General Internal Medicine, Department of Medicine, McMaster University, Hamilton, ON Canada

**Keywords:** Deep vein thrombosis, Guideline, Pulmonary embolism, Psychiatric in-patients, Thromboprophylaxis, Venous thromboembolism

## Abstract

**Introduction:**

Venous thromboembolism (VTE) is the leading cause of preventable hospital deaths. Adults hospitalised with psychiatric illness vary in their risk of VTE, and therefore in their likelihood of benefiting from thromboprophylaxis. There is a paucity of evidence-based practice guidelines addressing VTE prophylaxis for this population despite recognition of additional VTE risk factors in this population.

**Aim:**

To develop an evidence-based guideline on VTE prophylaxis for patients hospitalised with psychiatric illness using Grading of Recommendations, Assessment, Development and Evaluation (GRADE).

**Method:**

An international, multidisciplinary, guideline panel including clinical experts, methodologists, and a patient partner was recruited by invitation. Panelists were selected based on methodological and clinical expertise on this subject. Panel members were diverse in geography (from Ireland, the United Kingdom, France, and Canada), expertise and gender. The panel was composed of four advanced specialist psychiatric pharmacists, four consultant haematologists, four consultant psychiatrists, one advanced nurse practitioner in psychiatry, one advanced nurse practitioner in anticoagulation, a methodologist with expertise using GRADE, and a patient partner with lived experience of VTE. The panel prioritised two clinical questions and related population, interventions, outcomes, and secondary analyses according to their importance for patients. GRADE was used to assess certainty of evidence and to move from evidence to risk-stratified recommendations.

**Results:**

The panel made three recommendations: a strong recommendation against parenteral pharmacological prophylaxis for patients at low risk of VTE (moderate-certainty evidence); a conditional recommendation in favour of parenteral pharmacological prophylaxis in high-risk patients (low-certainty evidence); and a strong recommendation against graduated compression stockings in patients at high risk of VTE with a contraindication to parenteral pharmacological prophylaxis (low-certainty evidence).

**Conclusion:**

Clinicians should not use parenteral pharmacological prophylaxis in adults hospitalised with psychiatric illness at low risk of VTE; and should consider using parenteral pharmacological prophylaxis for high-risk adults with no contraindications. Graduated compression stockings are not recommended in high-risk patients when parenteral pharmacological prophylaxis is contraindicated. These GRADE- based recommendations offer one of the first evidence-based practice guidelines for thromboprophylaxis decisions in psychiatric in-patient settings.

**Supplementary Information:**

The online version contains supplementary material available at 10.1007/s11096-025-02072-1.

## Impact statements

- This is the first risk-stratified, evidence-based clinical practice guideline developed using GRADE to address VTE prophylaxis for adults hospitalised with psychiatric illness.
- This guideline has practice-changing potential for patients, pharmacists, and clinicians. It provides clear recommendations on the use of pharmacological prophylaxis and graduated compression stockings, incorporating multidisciplinary expertise and patient involvement in moving from evidence to recommendations.
- These recommendations are intended to support pharmacists, psychiatrists, and other clinicians in making informed, patient-centred decisions about VTE prophylaxis in psychiatric settings. They may also inform hospital policy, reduce practice variation, and highlight research priorities to strengthen evidence in this under-studied population.

## Introduction

Venous thromboembolism (VTE) is the third most common vascular disease worldwide with an incidence of one per 1000 annually among middle aged adults [1–3]. The risk increases with age and affects close to 10 per 1000 annually in those over the age of 75 years [1–3]. Up to 60% of all VTE events occur during or within 90 days of hospitalisation, making hospital-acquired thrombosis the leading cause of both VTE and of preventable hospital deaths [4]. VTE risk among hospitalised adults arises from the interplay of immobility, inflammation, vascular injury and hypercoagulability – factors that are common to many medical and surgical settings [1–4].

Psychiatric in-patients represent a heterogeneous population along this risk continuum [5–10]. While many are anticipated to be at relatively low baseline risk, several risk factors can increase the general VTE risk profile. These include markedly reduced mobility (e.g., due to illness, catatonia, sedation, physical restraint, periods of seclusion); the use of some antipsychotic medications associated with increased thrombotic risk; and older age, particularly among patients with dementia [5–10]. Collectively, these psychiatric-specific factors can overlap with or potentiate traditional medical VTE risk factors, underscoring the need for tailored risk assessment in this population.

In the absence of thromboprophylaxis, VTE risk varies from 0.3% among low-risk medical in-patients to 11% among high-risk medical in-patients [11]. Evidence regarding similar baseline risk estimates among psychiatric in-patients is of low or very low certainty and is limited to a small number of cohort studies [5–9]. Overall VTE rates in psychiatric hospitalised patients identified from observational studies range from 0.2% to 3.5%, and up to 12.5% for higher-risk adults [5–9].

Several international guidelines provide recommendations for medical and surgical in-patients, but evidence-based recommendations for VTE prophylaxis are lacking for adults hospitalised with psychiatric illness [1, 4, 10–12].

### Aim

This evidence-based guideline intends to support patients, pharmacists, clinicians and system-level decision-makers with trustworthy practice recommendations regarding VTE prophylaxis for adults hospitalised with psychiatric illness.

## Method

### Standards for trustworthy guidance

This guideline adheres to standards for trustworthy guidance from the Institute of Medicine (U.S.) and strives to meet standards of methodological rigour for guideline development [13, 14].

### Scope of the guideline

This guideline provides recommendations pertaining to adults aged 18 years and older and hospitalised with functional or organic psychiatric illness to medical or psychiatric hospitals in Ireland and the United Kingdom. Patients institutionalised in long-term care facilities or nursing homes, and those who are pregnant or within six weeks post-partum are outside the remit of this guideline.

The guideline focuses on prophylaxis and does not address treatment of VTE. The guideline focuses specifically on parenteral prophylaxis with low molecular weight heparin (LMWH) or fondaparinux, and on graduated compression stockings (GCS) for mechanical prophylaxis. Direct oral anticoagulants and intermittent pneumatic compression were considered but ultimately de-prioritised by the panel [4, 12, 15, 16].

### Guideline panel composition

A 16-member international, multidisciplinary, guideline panel including clinical experts, methodologists, and a patient partner was recruited by invitation. Panelists were selected based on methodological and clinical expertise on this subject. Panel members were diverse in geography (from Ireland, the United Kingdom, France, and Canada), expertise and gender. The panel was composed of four advanced specialist psychiatric pharmacists, four consultant haematologists, four consultant psychiatrists, one advanced nurse practitioner in psychiatry, one advanced nurse practitioner in anticoagulation, a methodologist with expertise applying the Grading of Recommendations, Assessment, Development and Evaluation (GRADE) approach, and a patient partner with lived experience of VTE.

Panel members disclosed possible conflicts of interest at the outset of the guideline development process; the clinical co-chairperson (AP) reviewed disclosures and confirmed no major financial conflicts of interest precluded participation in the guideline process. Development of this guideline was not funded (funding was limited to coverage of open-access fees for publication courtesy of St. John of God Research Foundation and St. John of God University Hospital Dublin, Ireland, with neither otherwise being directly involved in the guideline process), and panelists received no financial compensation for their participation. A methodologist experienced in the conduct of systematic reviews, guideline development and the use of GRADE [17, 18] (AA) co-led guideline discussions and the development of recommendations.

### Formulation of clinical questions

The panel ultimately prioritised two clinical questions and related population, interventions, outcomes, and secondary analyses according to their importance for patients [1, 19–21].

*PICO 1: Among adults hospitalised with psychiatric illness, should parenteral anticoagulation (low-molecular weight heparin (LMWH) or fondaparinux), compared to no parenteral anticoagulation, be used for VTE prophylaxis?*

*PICO 2: Among adults hospitalised with psychiatric illness who are at high risk of VTE but in whom pharmacological prophylaxis is contraindicated, should graduated compression stockings (GCS), compared to no GCS, be used for mechanical VTE prophylaxis?*

### Identification of evidence

To inform baseline risk estimates used across prioritised outcomes, an electronic literature search was conducted using PubMed, Ovid, Embase, CINAHL, Web of Science, APA PsycInfo, and Scopus, from inception to October 2024. Search terms included: deep vein thrombosis; venous thromboembolism; pulmonary embolism; psychiatry; mental health; in-patient, incidence and rate. Cohort studies reporting on the incidence of VTE outcomes in hospitalised psychiatric patients were deemed eligible (see Appendix 1 for PRISMA flow diagram). The guideline co-chair (AP) screened identified hits for inclusion, and for eligible studies, conducted data extraction. The guideline methods co-chair (AA) cross-checked all extracted data.

To further inform baseline risk estimates for VTE outcomes and to inform estimates for other prioritised outcomes, a second informal literature search was conducted using the websites of professional societies involved in guideline development regarding VTE prophylaxis [1, 4, 19–21].

To address the second clinical question, we relied on two Cochrane reviews comparing the use of GCS to no GCS for VTE prophylaxis in hospitalised medical, surgical, and post-stroke patients [22, 23]. The clinical co-chair (AP) reviewed randomised trials from the two reviews for eligibility, and pooled study results from the included trials [22, 23]. The panel was aware of two landmark multi-centre studies relevant to the second clinical question that warranted updating of the existing reviews. Both trials compared GCS versus no GCS, the former in surgical patients, and the latter in stroke patients [24, 25]. We therefore additionally included and extracted data from the two studies. Extracted data across studies was partially cross-checked for accuracy by the methods co-chair (AA). Pooled analyses and forest plots were generated using Cochrane’s Review Manager (RevMan) version 9.13.0 [26] (see Appendix 2). Risk of bias ratings for studies identified from the Cochrane reviews were adopted directly from the reviews [22, 23], and additional risk of bias assessments were conducted in duplicate (AP, AA) using the Cochrane risk of bias tool [27] for the additional studies added [24, 25, 28]. We conducted a subgroup analysis for studies that were deemed to be low risk of bias across domains versus those with concerns regarding possible bias.

Forest plots were generated only for the second clinical question, given the addition of new trial data. For the first question, we adopted relative treatment effects directly from existing American Society of Hematology 2018 prophylaxis guidelines and associated systematic reviews and meta-analyses [1], and new forest plots were deemed unnecessary.

### Approach to risk stratification and estimation of baseline risks

The panel judged that benefits and harms of thromboprophylaxis were likely to vary according to adults’ baseline risk of venous thrombotic events. Adults with higher baseline risks of such events were generally anticipated to derive greater benefit from prophylaxis. Recommendations were therefore made for two risk groups based on their risk of VTE events. Based on existing prognostic models and risk prediction models, risk factors that are likely to result in an adult hospitalised with psychiatric illness being deemed high risk for VTE include but are not limited to: significantly reduced mobility relative to normal, previous VTE, or thrombophilic conditions [4, 11, 15, 16, 29].

Baseline risks adopted for the guideline are presented in Table 1. Estimates for both groups were informed by a review of cohort studies [5–9], evaluating the incidence of VTE outcomes among psychiatric patients and by outputs from commonly used VTE risk prediction models [4, 11, 15, 16, 29]. The panel also considered baseline risks used in parallel VTE prophylaxis guidelines including those produced by the American Society of Haematology when making its judgements [1].

**Table 1:** Estimated baseline risks used to inform absolute treatment effect estimates:

Outcomes	Low risk	High risk
All-cause mortality	0.5% (5 per 1000)	2% (20 per 1000)
Symptomatic pulmonary embolism	0.2% (2 per 1000)	2% (20 per 1000)
Symptomatic proximal deep vein thrombosis	0.2% (2 per 1000)	2% (20 per 1000)
Symptomatic distal deep vein thrombosis	0.4% (4 per 1000)	4% (40 per 1000)
Major bleeding	0.5% (5 per 1000)	
Gastrointestinal bleeding	1% (10 per 1000)	
Heparin-induced thrombocytopenia	0%	

While the risk of bleeding was anticipated to vary across adults, the panel surmised that these risks were unlikely to systematically differ between adults at low and at high risk of VTE in the absence of pharmacological prophylaxis. A single set of unstratified baseline risks were considered from the American Society of Haematology VTE prophylaxis guidelines [1] and used to estimate baseline risks for these outcomes.

In clinical decision-making, an individualised assessment of bleeding risk should accompany evaluation of VTE risk. Medications that may increase bleeding risk include concomitant use of antiplatelet medications, non-steroidal anti-inflammatory drugs and serotonin reuptake inhibitor antidepressants (e.g. selective serotonin reuptake inhibitors (SSRIs) and serotonin and noradrenaline reuptake inhibitors (SNRIs)) [29–31]. While specific data quantifying these risks among adults hospitalised with psychiatric illness are lacking, clinicians should be mindful of these considerations when determining suitability for pharmacological prophylaxis.

### Estimation of treatment effects

Data regarding the effects of pharmacological prophylaxis and of GCS on outcomes for adults hospitalised with psychiatric illness was unavailable based on informal literature reviews and on panel discussions. In the absence of such data, the panel relied on available data from medical and surgical in-patient populations. The panel acknowledged that differences in treatment effects between these patient populations and psychiatric in-patients were plausible but unlikely.

To address the first prioritised question, the panel therefore relied on relative treatment effect estimates from the 2018 American Society of Hematology VTE prophylaxis guidelines for medical in-patients [1]. The panel perceived that the relative effects of treatments was likely to be similar. To address the second question, the panel relied on treatment effect estimates from its pooled analysis, summarised above [22–25]. Given the residual possibility that treatments may perform differently among psychiatric in-patients than the populations in the included studies, the panel considered issues regarding indirectness when judging certainty of the evidence and making recommendations.

### Moving from evidence to recommendations

Both prioritised questions relied on randomised trial evidence. Therefore, when rating certainty of evidence for a given outcome using GRADE, ratings began as high certainty and could be downgraded for limitations with regards to imprecision, inconsistency, risk of bias, indirectness, and publication bias [17, 18].

Recommendations made using GRADE were either strong or conditional in terms of their strength, and in favour or against treatment in terms of direction (Appendix 3) [17, 18]. The panel considered the balance of benefits, harms and burdens of treatments, certainty of evidence informing these effects and the assumed values and preferences of adults hospitalised with psychiatric illness. Secondarily, they incorporated resource use, acceptability, feasibility, and equity considerations when making judgements. For the three recommendations made, these secondary considerations did not weigh heavily on decision-making.

The guideline panel attended four online meetings via Microsoft Teams (7th January, 11th March, 29th April and 27th May 2025). Clinical and methods co-chairs (AP and AA) co-facilitated discussions in all meetings. The panel adopted a consensus-based approach to moving from evidence to recommendations, whereby discussion and informal voting was used to determine the strength and direction of recommendations. Unanimous or near-unanimous consensus was achieved for all recommendations provided.

### Values and preferences of adults hospitalised with psychiatric illness

In the absence of credible evidence regarding the values and preferences of adults hospitalised with psychiatric illness to inform recommendations, the panel relied on their own judgements regarding how average patients were anticipated to make decisions. These judgements were vetted by the patient partner on the panel.

The panel agreed that most well-informed adults hospitalised with psychiatric illness would be inclined to accept therapy when faced with moderate or high certainty of important benefits, and moderate or high certainty of little or no increased risk of harms. However, most well-informed adults were anticipated to be disinclined from accepting therapy when faced with moderate or high certainty of little or no benefit, regardless of harms; or moderate or high certainty of potential patient-important harms.

Additional input from the patient partner suggested that adults hospitalised with psychiatric illness are likely to place much greater value on a reduction in VTE risk than on a comparable increase in bleeding-related harms and treatment burdens; and are likely to view DVT and PE as relatively equal in importance. Our patient partner advocated that all patients hospitalised with psychiatric illness at high risk of VTE should have access to VTE risk assessment.

The panel selected two minimal important difference (MID) thresholds for patient-important effects based on consensus *a priori*: 0.5% (5 per 1000) for all-cause mortality and 1% (10 per 1000) for all other outcomes.

### Ethics approval

No ethics approval was required as no primary data was collected, and this is a practice guideline focused on development of recommendations for practice.

## Results

Recommendations are summarized in Table 2 and are stratified by risk of VTE. The panel acknowledged that there are different VTE risk assessment models in use [12, 15, 16] and recommended VTE risk assessments be completed according to local or national protocols.

**Table 2:** Summary of recommendations

Population	Intervention	Recommendation
Low risk of VTE	Prophylactic parenteral anticoagulation	***Recommendation #1:*** In adults hospitalised with psychiatric illness and at *low* risk of VTE, we *recommend against* using prophylactic parenteral anticoagulation (strong recommendation against; moderate-certainty evidence)
High risk of VTE	Prophylactic parenteral anticoagulation	***Recommendation #2:*** In adults hospitalised with psychiatric illness and at high risk of VTE, we *suggest in* favour of prophylactic parenteral anticoagulation (conditional recommendation in favour; low-certainty evidence)
High risk of VTE and in whom pharmacological thromboprophylaxis is contraindicated	Graduated compression stockings	***Recommendation #3:*** In adults hospitalised with psychiatric illness who are at high risk of VTE and in whom pharmacological thromboprophylaxis is contraindicated, we *recommend against* using GCS (strong recommendation against; low-certainty evidence)

**Recommendation #1:** In adults hospitalised with psychiatric illness and at *low* risk of VTE, we *recommend against* using prophylactic parenteral anticoagulation (strong recommendation against; moderate-certainty evidence).

*Evidence –* see Table 3. In adults at low risk, parenteral prophylaxis has little or no effect on symptomatic proximal DVT (1 fewer per 1000 adults, 95% CI 2 fewer to 1 more) and symptomatic distal DVT (1 fewer per 1000, 3 fewer to 9 more) (both high certainty). Parenteral prophylaxis probably has little or no effect on all-cause death (0 fewer per 1000 adults, 0 fewer to 0 more) and symptomatic PE (1 fewer per 1000, 1 fewer to 0 more) (both moderate certainty). Parenteral prophylaxis has a trivial effect on risk of HIT (mean difference 2 more per 1000; high certainty) and probably has a trivial effect on major bleeding (2 more per 1000, 95% CI 1 fewer to 9 more; moderate certainty). Very low certainty evidence informed effects on gastrointestinal bleeding.

**Table 3:** Summary of findings: parenteral prophylaxis in low-risk adults

Outcome Timeframe	Study results and measurements	Absolute effect estimates		Certainty of the evidence	Summary
		No parenteral anticoagulation	Prophylactic parenteral anticoagulation		
All-cause death 90 days	Relative risk: 0.97 (CI 95% 0.91—1.04) Based on data from 49,002 participants in 21 studies	**5** per 1000	**5** per 1000	**Moderate** Due to serious risk of bias and concerns regarding indirectness ^a, b^	Parenteral anticoagulation probably has little or no effect on all-cause death
		Difference: **0 fewer per 1000** (CI 95% 0 fewer—0 fewer)			
Symptomatic pulmonary embolism 90 days	Relative risk: 0.59 (CI 95% 0.45—0.78) Based on data from 25,687 participants in 13 studies	**2** per 1000	**1** per 1000	**Moderate** Due to serious risk of bias and concerns regarding indirectness ^a, b^	Parenteral anticoagulation probably has little or no effect on symptomatic pulmonary embolism
		Difference: **1 fewer per 1000** (CI 95% 1 fewer—0 more)			
Symptomatic proximal deep vein thrombosis 90 days	Relative risk: 0.28 (CI 95% 0.06—1.37) Based on data from 3706 participants in 1 study	**2** per 1000	**1** per 1000	**High** Concerns regarding indirectness ^a^	Parenteral anticoagulation has little or no effect on symptomatic proximal deep vein thrombosis
		Difference: **1 fewer per 1000** (CI 95% 2 fewer—1 more)			
Symptomatic distal deep vein thrombosis 90 days	Relative risk: 0.75 (CI 95% 0.17—3.34) Based on data from 3706 participants in 1 study	**4** per 1000	**3** per 1000	**High** Concerns regarding indirectness ^a^	Parenteral anticoagulation has little or no effect on symptomatic distal deep vein thrombosis
		Difference: **1 fewer per 1000** (CI 95% 3 fewer—9 more)			
Major bleeding 90 days	Relative risk: 1.48 (CI 95% 0.81—2.71) Based on data from 30,761 participants in 16 studies	**5** per 1000	**7** per 1000	**Moderate** Due to serious risk of bias and concerns regarding indirectness ^a, b^	Parenteral anticoagulation probably has little or no effect on major bleeding
		Difference: **2 more per 1000** (CI 95% 1 fewer—9 more)			
Gastrointestinal bleeding 90 days	Relative risk: 2.61 (CI 95% 0.36—18.86) Based on data from 185 participants in 2 studies	**10** per 1000	**26** per 1000	**Very low** Due to serious risk of bias, very serious imprecision and concerns regarding indirectness ^a, b, c^	The effect of parenteral anticoagulation on gastrointestinal bleeding is very uncertain
		Difference: **16 more per 1000** (CI 95% 6 fewer—179 more)			
Heparin-induced thrombocytopenia 90 days	Based on data from 12,577 participants in 3 studies	**0** per 1000	**2** per 1000	**High** Concerns regarding indirectness ^a^	Parenteral anticoagulation has little or no effect on heparin-induced thrombocytopenia
		Difference: **2 more per 1000**			

^a^Concerns regarding indirectness were noted, given included studies were conducted in a population that was different than the target population of the guideline

^b^Rated down for risk of bias due to “unclear random sequence generation and allocation concealment across majority of trials, and lack of blinding of participants and study personnel as well as unclear blinding of outcome assessors” as per American Society of Hematology 2018 guidelines for management of venous thromboembolism

^c^Rated down for very serious imprecision due to very wide confidence intervals crossing the panel’s pre-specified decision threshold (minimal important difference threshold)

Population: Low-risk adults hospitalised with psychiatric illness

Intervention: Prophylactic parenteral anticoagulation

Comparator: No parenteral anticoagulation

*Understanding the recommendation*—Given moderate to high certainty of little or no benefit with regards to survival and VTE risk, all or almost all low-risk adults hospitalised with psychiatric illness were anticipated to be disinclined to receive parenteral prophylactic anticoagulation, justifying a strong recommendation against therapy.

*Practical information*—Given the strong recommendation against, practical considerations were judged to be less relevant here.

**Recommendation #2:** In adults hospitalised with psychiatric illness and at high risk of VTE, we *suggest in* favour of prophylactic parenteral anticoagulation (conditional recommendation in favour; low-certainty evidence).

*Evidence –* see Table 4. In adults at high risk, parenteral prophylaxis probably has little or no effect on all-cause death (1 fewer per 1000, 95% CI 2 fewer to 1 more; moderate certainty) and may have a trivial effect on symptomatic PE (8 fewer per 1000, 11 fewer to 4 fewer; low certainty). Parenteral prophylaxis probably reduces the risk of symptomatic proximal DVT (14 fewer per 1000, 19 fewer to 7 more; moderate certainty) and may reduce the risk of symptomatic distal DVT (10 fewer per 1000, 33 fewer to 94 more; low certainty). Effects on bleeding outcomes and HIT are the same as for recommendation #1 above.

**Table 4:** Summary of findings: parenteral prophylaxis in high-risk adults

Outcome Timeframe	Study results and measurements	Absolute effect estimates		Certainty of the evidence	Summary
		No parenteral anticoagulation	Prophylactic parenteral anticoagulation		
All-cause death 90 days	Relative risk: 0.97 (CI 95% 0.91—1.04) Based on data from 49,002 participants in 21 studies	**20** per 1000	**19** per 1000	**Moderate** Due to serious risk of bias and concerns regarding indirectness ^a, b^	Parenteral anticoagulation probably has little or no effect on all-cause death
		Difference: **1 fewer per 1000** (CI 95% 2 fewer—1 more)			
Symptomatic pulmonary embolism 90 days	Relative risk: 0.59 (CI 95% 0.45—0.78) Based on data from 25,687 participants in 13 studies	**20** per 1000	**12** per 1000	**Low** Due to serious risk of bias, serious imprecision and concerns regarding indirectness ^a, b, c^	Parenteral anticoagulation may have little or no effect on symptomatic pulmonary embolism
		Difference: **8 fewer per 1000** (CI 95% 11 fewer—4 fewer)			
Symptomatic proximal deep vein thrombosis 90 days	Relative risk: 0.28 (CI 95% 0.06—1.37) Based on data from 3706 participants in 1 study	**20** per 1000	**6** per 1000	**Moderate** Due to serious imprecision and concerns regarding indirectness ^a, c^	Parenteral anticoagulation probably reduces symptomatic proximal deep vein thrombosis
		Difference: **14 fewer per 1000** (CI 95% 19 fewer—7 more)			
Symptomatic distal deep vein thrombosis 90 days	Relative risk: 0.75 (CI 95% 0.17—3.34) Based on data from 3706 participants in 1 study	**40** per 1000	**30** per 1000	**Low** Due to very serious imprecision and concerns regarding indirectness ^a, d^	Parenteral anticoagulation may reduce symptomatic distal deep vein thrombosis
		Difference: **10 fewer per 1000** (CI 95% 33 fewer—94 more)			
Major bleeding 90 days	Relative risk: 1.48 (CI 95% 0.81—2.71) Based on data from 30,761 participants in 16 studies	**5** per 1000	**7** per 1000	**Moderate** Due to serious risk of bias and concerns regarding indirectness ^a, b^	Parenteral anticoagulation probably has little or no effect on major bleeding
		Difference: **2 more per 1000** (CI 95% 1 fewer—9 more)			
Gastrointestinal bleeding 90 days	Relative risk: 2.61 (CI 95% 0.36—18.86) Based on data from 185 participants in 2 studies	**10** per 1000	**26** per 1000	**Very low** Due to serious risk of bias, very serious imprecision and concerns regarding indirectness ^a, b, d^	The effect of parenteral anticoagulation on gastrointestinal bleeding is very uncertain
		Difference: **16 more per 1000** (CI 95% 6 fewer—179 more)			
Heparin-induced thrombocytopenia 90 days	Based on data from 12,577 participants in 3 studies	**0** per 1000	**2** per 1000	**High** Concerns regarding indirectness ^a^	Parenteral anticoagulation has little or no effect on heparin-induced thrombocytopenia
		Difference: **2 more per 1000**			

^a^Concerns regarding indirectness was noted, given included studies were conducted in a population that was different than the target population of the guideline

^b^Rated down for risk of bias due to “unclear random sequence generation and allocation concealment across majority of trials, and lack of blinding of participants and study personnel as well as unclear blinding of outcome assessors” as per American Society of Haematology 2018 guidelines for management of venous thromboembolism

^c^Rated down for serious imprecision due to wide confidence intervals crossing the panel’s pre-specified decision threshold (minimal important difference threshold)

^d^Rated down for very serious imprecision due to very wide confidence intervals crossing the panel’s pre-specified decision threshold (minimal important difference threshold)

Population: High-risk adults hospitalised with psychiatric illness

Intervention: Prophylactic parenteral anticoagulation

Comparator: No parenteral anticoagulation

*Understanding the recommendation*—Given low to moderate certainty of important reductions in the risks of symptomatic proximal and distal DVT, moderate-certainty evidence of little or no increased risk of major bleeding, and no other substantial harms or burdens anticipated, most high-risk adults were anticipated to be inclined to receive parenteral prophylactic anticoagulation. However, given the certainty of evidence supporting outcomes demonstrating benefit, variability in risk profiles of individuals at high risk and residual possibility of bleeding-related harms (particularly given very low certainty evidence for gastrointestinal bleeding), the panel judged that a reasonable proportion of adults may be disinclined from receiving therapy. This variability in anticipated decision-making justified a conditional recommendation in favour of therapy.

*Practical information*—Prophylactic doses of low molecular weight heparin given by subcutaneous injection are generally used as first-line management for VTE prophylaxis. This should be tailored to the individual, as kidney function and the weight of the patient need to be considered when selecting an appropriate dose.

The panel acknowledged issues with capacity and/or consent for VTE prophylaxis may be a consideration in some acutely unwell adults hospitalised with psychiatric illness.

Efforts should be prioritised to increase awareness regarding clinical signs and symptoms of DVT and PE among medical and nursing staff providing care on psychiatric units, and among all adults hospitalised with psychiatric illness.

General VTE reduction measures for all hospitalised patients include adequate hydration, early mobilisation, and leg exercises [4].

When initiating prophylactic anticoagulation, clinicians should assess concurrent medications and bleeding risk factors. Clinicians should consider the addition of proton pump inhibitors as appropriate to mitigate the risk of gastrointestinal bleeding, particularly in patients receiving SSRI or SNRI antidepressants [29–31].

**Recommendation #3:** In adults hospitalised with psychiatric illness who are at high risk of VTE and in whom pharmacological thromboprophylaxis is contraindicated, we *recommend against* using GCS (strong recommendation against; low-certainty evidence).

*Evidence –* see Table 5. In adults at high risk in whom pharmacological thromboprophylaxis is contraindicated, GCS have little or no effect on symptomatic proximal DVT (3 fewer per 1000, 95% CI 9 fewer to 6 more; high certainty). GCS probably have a trivial effect on all-cause death (2 more per 1000, 3 fewer to 8 more; moderate certainty). Low-certainty evidence showed possibly little or no effect of GCS on symptomatic distal DVT (2 more per 1000, 18 fewer to 40 more) and symptomatic PE (7 fewer per 1000, 13 fewer to 5 more). Adverse events and discomfort with GCS each occurred in approximately 5% of adults in included trials (high certainty).

**Table 5:** Summary of findings: prophylaxis with graduated compression stockings in high-risk adults in whom pharmacological prophylaxis is contraindicated

Outcome Timeframe	Study results and measurements	Absolute effect estimates		Certainty of the evidence	Summary
		No GCS	GCS		
All-cause death 90 days	Relative risk: 1.11 (CI 95% 0.87—1.41) Based on data from 4376 participants in 2 studies	**20** per 1000	**22** per 1000	**Moderate** Due to serious imprecision and concerns regarding indirectness ^a, b^	Graduated compression stockings probably have little or no effect on all-cause death
		Difference: **2 more per 1000** (CI 95% 3 fewer—8 more)			
Symptomatic proximal DVT 90 days	Relative risk: 0.84 (CI 95% 0.54—1.3) Based on data from 2518 participants in 1 study	**20** per 1000	**17** per 1000	**High** concerns regarding indirectness ^a^	Graduated compression stockings have little or no effect on symptomatic proximal DVT
		Difference: **3 fewer per 1000** (CI 95% 9 fewer—6 more)			
Symptomatic distal DVT 90 days	Relative risk: 1.06 (CI 95% 0.56—2.01) Based on data from 2518 participants in 1 study	**40** per 1000	**42** per 1000	**Low** Due to very serious imprecision ^c^	Graduated compression stockings may have little or no effect on symptomatic distal DVT
		Difference: **2 more per 1000** (CI 95% 18 fewer—40 more)			
Symptomatic pulmonary embolism 90 days	Relative risk: 0.64 (CI 95% 0.33—1.25) Based on data from 4376 participants in 2 studies	**20** per 1000	**13** per 1000	**Low** Due to serious indirectness and serious imprecision ^b, d^	Graduated compression stockings may have little or no effect on symptomatic pulmonary embolism
		Difference: **7 fewer per 1000** (CI 95% 13 fewer—5 more)			
Adverse events 90 days	Narrative summary Based on data from 2 studies			**High**	54 per 1000 adults (5.4%) using graduated compression stockings encountered related adverse events in included trials. 47 per 1000 adults (4.7%) using stockings reported related discomfort.

^a^Concerns regarding indirectness were noted, given included studies were conducted in a population that was different than the target population of the guideline

^b^Rated down for serious imprecision due to wide confidence intervals crossing the panel’s pre-specified decision threshold (minimal important difference threshold)

^c^Rated down for very serious imprecision due to very wide confidence intervals crossing the panel’s pre-specified decision threshold (minimal important difference threshold)

^d^Rated down for serious indirectness, given included studies were conducted in a population that was different than the target population of the guideline; and studies included symptomatic and asymptomatic events and involved variable follow-up durations differing from the target timeframe for the guideline

Population: High-risk adults hospitalised with psychiatric illness in whom pharmacological prophylaxis contraindicated

Intervention: Graduated compression stockings

Comparator: No graduated compression stockings

*Understanding the recommendation*—Given little or no effect on survival and prioritised VTE outcomes and the possibility of adverse events and stocking-related discomfort, all or almost all well-informed adults were anticipated to be disinclined from receiving GCS. The panel additionally considered that GCS may pose a ligature risk and a risk of self-harm in this patient population, further supporting a strong recommendation against therapy.

*Practical information*— Given the strong recommendation against, practical considerations were judged to be less relevant here.

## Discussion

To our knowledge, this is one of the first clinical practice guidelines to be submitted for national-level implementation, developed using GRADE, which provides recommendations regarding VTE risk reduction in adults hospitalised with psychiatric illness. The guideline draws on multidisciplinary expertise free of major financial and intellectual conflicts of interest; incorporates the views of a patient partner with lived experience and the perceived values and preferences of average well-informed adults hospitalised with psychiatric illness; and bases recommendations on best available evidence to inform baseline risks and treatment effects across prioritised patient-important outcomes.

Limitations of our guideline are primarily reflective of limitations in available evidence and reflect an opportunity for ongoing improvement in evidence generation and subsequent guideline endeavors. First, in the absence of a validated VTE risk assessment model or tool for adults with psychiatric illness, we risk-stratify adults as being in one of two VTE risk groups. Development of a validated tool will help to reduce currently existing practice variation across institutions in terms of risk assessment protocols and policies [10, 12]. Second, baseline risk estimates across key patient-important outcomes were primarily informed by low-certainty cohort study evidence. Third, evidence regarding patient values and preferences for the target population, and thresholds for treatment effects they are likely to perceive as important or unimportant, are lacking. Fourth, credible evidence regarding treatment effects for both interventions of interest were unavailable in psychiatric in-patient populations, leaving decision-makers and guideline developers to make inferences based on indirect evidence. A further limitation in available evidence was that many studies did not differentiate between symptomatic and asymptomatic VTE events and used variable methods for diagnosis of VTE.

The panel therefore identified numerous residual uncertainties in available evidence and priorities for future research:

- Development and validation of a VTE risk assessment model or tool in adults hospitalised with psychiatric illness.
- Credible evidence regarding individualised bleeding risk estimation for adults hospitalised with psychiatric illness.
- Rigorous observational study data to inform risk-stratified baseline risk estimates across prioritised patient-important outcomes.
- Credible evidence regarding the values and preferences of average well-informed adults hospitalised with psychiatric illness.
- Credible randomised trial evidence regarding the effects of prophylactic parenteral anticoagulation and GCS among adults hospitalised with psychiatric illness.
- Clear reporting of symptomatic VTE outcomes and use of consistent diagnostic methods.
- Feasibility, tolerability, acceptability and effectiveness of intermittent pneumatic compression as mechanical prophylaxis for high-risk adults hospitalised with psychiatric illness.

## Conclusion

VTE risk assessment and prophylaxis should be tailored to the needs of the individual patient and underpinned by evidence-based recommendations. We strongly recommend against the use of prophylactic parenteral anticoagulation in low-risk adults and conditionally recommend in favour of its use in high-risk adults. This is the first guideline using GRADE to provide evidence-informed recommendations for this patient population. These recommendations are intended to complement existing hospital thromboprophylaxis protocols and may inform policies nationally or internationally.

## Supplementary Information

Below is the link to the electronic supplementary material.

Supplementary file1 (DOCX 275 KB)

## Data Availability

All data generated to inform this practice guideline is included in this publication.
