# Supplementary material for: Venous thromboembolism prophylaxis in adults hospitalised for psychiatric illness: an evidence-based clinical practice guideline developed using GRADE
Source: Int J Clin Pharm. 2026 Jan 16;48(3):777–88. doi: 10.1007/s11096-025-02072-1 (PMC13176174; doi:10.1007/s11096-025-02072-1)
Supplement: Supplementary file 1 — Supplementary file1 (DOCX 275 KB) [file 11096_2025_2072_MOESM1_ESM.docx]

**Venous thromboembolism prophylaxis in adults hospitalised for psychiatric illness: an evidence-based clinical practice guideline developed using GRADE.**

**International Journal of Clinical Pharmacy.**

Purcell A ^1,2^, Ní Áinle F ^3,4^, Hunt BJ ^5^, Delluc A ^6^, Roberts L ^7^, Jenkinson J ^8-10^, Hoblyn J ^1,4^, Keating D ^1,2^, Carolan A ^1,2^, Roche E ^1,11,12^, Morgan K ^13^, Duffy R ^4, 14^, Garvey S ^15^, Flood J ^16^, O Neill A ^17^, Agarwal A ^18-20^

^1^ St John of God University Hospital, Dublin, Ireland; ^2^ School of Pharmacy and Biomolecular Science, Royal College of Surgeons, Dublin, Ireland; ^3^ National Clinical Lead, National Clinical Programme in Venous Thromboembolism, Health Services Executive, Dr Steeven's Hospital, Dublin, Ireland; ^4^ School of Medicine, University College Dublin, Ireland; ^5^ Department of Thrombosis & Haemostasis, Kings Healthcare Partners, London, UK; ^6^ University of Ottawa, Faculty of Medicine, Department of Medicine, Division of Hematology, Canada; ^7^ Department of Haematological Medicine, King's College Hospital NHS Foundation Trust, UK; ^8^ Surrey and Borders Partnership NHS Foundation Trust, UK; ^9^ Department of Brain Sciences Imperial College London ; ^10^ Royal College of Psychiatrists UK, Faculty of Old Age Psychiatry; ^11^ Cluain Mhuire Community Mental Health Service, Dublin, Ireland; ^12^ School of Medicine, RCSI University of Medicine and Health Sciences, Dublin, Ireland; ^13^ Pennine Care NHS Foundation Trust UK; ^14^ Rotunda Hospital, Dublin, Ireland; ^15^ Beaumont Hospital, Dublin, Ireland; ^16^ Mental Health Services for Older People; North Dublin, Ireland; ^17^ Patient partner; ^18^ Division of General Internal Medicine, Department of Medicine, University of Alberta, Edmonton, Alberta; ^19^ Department of Health Research Methods, Evidence and Impact, McMaster University, Hamilton, Ontario, Canada; ^20^ Division of General Internal Medicine, Department of Medicine, McMaster University, Hamilton, Ontario, Canada.

**Corresponding author:**

arnav.agarwal@ualberta.ca

**Appendix 1: Prisma Flow Diagram: Venous thromboembolism incidence in patients hospitalised for psychiatric illness:**

Studies from databases/registers **(n = 3796)**

Embase (n = 1224)

MEDLINE (n = 747)

Scopus (n = 654)

Web of Science (n = 606)

CINAHL (n = 298)

CENTRAL (n = 154)

PsycINFO (n = 112)

Cochrane Reviews (n = 1)

**Identification**

Studies excluded **(n = 2314)**

Titles and abstracts screened **(n = 2336)**

Duplicates removed **(n = 1460)**

Studies excluded **(n = 17)**

Wrong outcomes (n = 1)

Wrong study design (n = 6)

Wrong patient population (n = 10)

Full-text articles assessed for eligibility **(n = 22)**

**Screening**

Studies included in review **(n = 5)**

**Included**

**Appendix 2: Forest plots PICO 2 Graduated Compression Stockings versus no Graduated Compression Stockings:**

**Subgroup analysis: symptomatic events and low risk of bias studies**

**1.All-cause mortality analysis:**


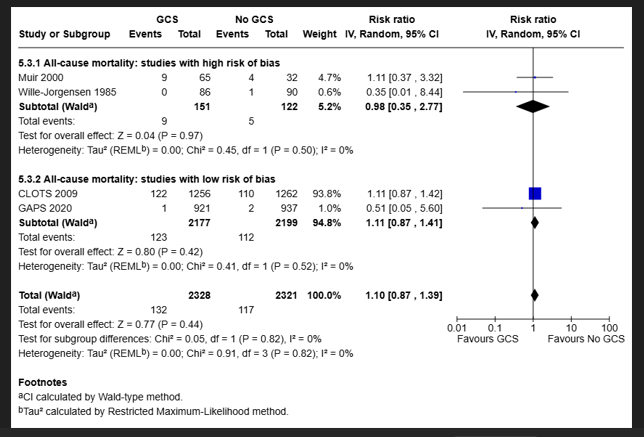


**2. Symptomatic proximal DVT analysis:**


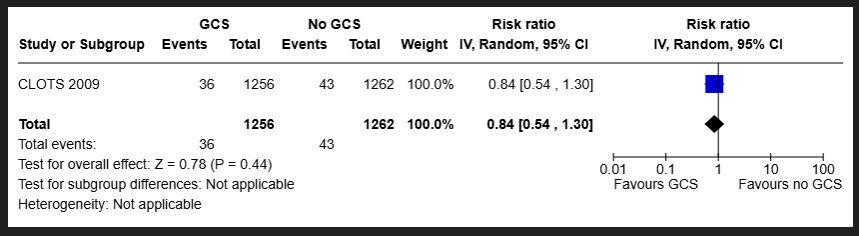


**3. Symptomatic distal DVT analysis:**


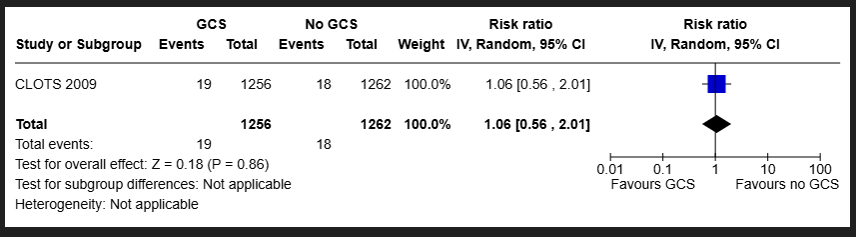


**4. Symptomatic Pulmonary Embolism analysis:**

**
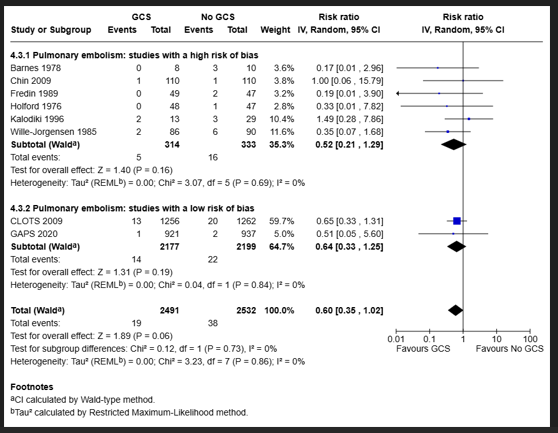
**

**Appendix 3: GRADE Strong and Conditional Recommendations:**

**Strong Recommendations:**

• For patients: most individuals in this situation would want the recommended course

of action, and only a small proportion would not.

• For clinicians: most individuals should follow the recommended course of action.

Formal decision aids are not likely to be needed to help individual patients make

decisions consistent with their values and preferences.

• For policy makers: the recommendation can be adopted as policy in most situations.

Adherence to this recommendation according to the guideline could be used as a

quality criterion or performance indicator.

• For researchers: the recommendation is supported by credible research or other

convincing judgments that make additional research unlikely to alter the

recommendation. On occasion, a strong recommendation is based on low or very

low certainty in the evidence. In such instances, further research may provide

important information that alters the recommendations.

**Conditional Recommendations:**

• For patients: the majority of individuals in this situation would want the suggested

course of action, but many would not. Decision aids may be useful in helping

patients to make decisions consistent with their individual risks, values, and

preferences.

• For clinicians: different choices will be appropriate for individual patients, and

clinicians must help each patient arrive at a management decision consistent with

the patient's values and preferences. Decision aids may be useful in helping

individuals to make decisions consistent with their individual risks, values, and

preferences.

• For policy makers: policy making will require substantial debate and involvement of

various stakeholders. Performance measures about the suggested course of action

should focus on whether an appropriate decision-making process is duly

documented.

• For researchers: this recommendation is likely to be strengthened (for future

updates or adaptation) by additional research. An evaluation of the conditions and

criteria (and the related judgments, research evidence, and additional

considerations) that determined the conditional (rather than strong)

recommendation will help to identify possible research gaps.
